# The capsule was never just a capsule: what islet encapsulation is for, now that immunoisolation is no longer its job

**DOI:** 10.3389/fbioe.2026.1943061

**Published:** 2026-10-08

**Authors:** Antonio Citro, Lorenzo Piemonti

**Affiliations:** 1 Diabetes Research Institute (DRI), IRCCS Ospedale San Raffaele, Milan, Italy; 2 Università Vita-Salute San Raffaele, Milan, Italy

**Keywords:** islet encapsulation, immunoisolation, hypoimmune cells, gene editing, β-cell replacement, containment and retrievability, stem-cell-derived islets, type 1 diabetes

## Abstract

For more than four decades, islet encapsulation has been defined by a single objective: to immunoprotect transplanted cells while preserving glucose sensing and insulin delivery, thereby eliminating systemic immunosuppression. No passive device has yet achieved that objective with reproducible, durable and clinically meaningful efficacy. The central limitation is not simply the absence of an optimal polymer, but an over-constrained architecture in which immune exclusion, molecular transport, therapeutic cell dose, host integration, mechanical durability and retrieval must be solved simultaneously. The clinical contrast between sealed and vascularly accessible devices makes this coupling visible: closure preserves immunological separation but intensifies transport and foreign-body constraints; opening improves tissue integration but restores immune access. Hypoimmune cell engineering now changes the allocation of these functions. Gene-edited allogeneic islet cells have survived without a capsule or systemic immunosuppression in immunocompetent primates and, to date, in one human recipient from a single programme, although therapeutic sufficiency, scalability, independent replication and long-term safety remain unresolved. We argue that this development should not make encapsulation obsolete, but should redefine its target product profile. When immune protection is increasingly engineered into the cell product, the device can be optimised for the functions that remain uniquely physical: containment, vascular integration, complete retrievability and layered safety. This shifts the biomaterials problem from molecular immunoisolation to a low-transport-resistance, defect-controlled and mechanically durable containment platform for proliferative, immune-evasive cell therapies.

## 1 Introduction

In 1980, Lim and Sun showed that microencapsulated islets could restore glycaemic control in diabetic rodents, establishing immunoisolation as an apparently elegant route to cell therapy: glucose and insulin would cross a selectively permeable membrane, whereas the immune system would not (Lim and Sun, 1980). Forty-six years later, the concept remains compelling; a reproducible therapy has not followed.

Its persistence is understandable: encapsulation promised to address allogeneic rejection, recurrent autoimmunity, immunosuppression toxicity and cell-source flexibility within a single engineering solution (Orive et al., 2003; Scharp and Marchetti, 2014; Desai and Shea, 2017).

For four decades the predominant response to disappointing clinical results has been iterative material optimisation—purified alginates, thinner membranes, more durable polymers—on the premise that immunoisolation is principally a materials-chemistry problem (Calafiore et al., 2006; Vegas et al., 2016a; Desai and Shea, 2017). This Perspective advances a different, systems-level reappraisal. First, passive immunoisolation has remained clinically limited not for want of the right polymer but because immunity, transport, geometry, dose, host response, manufacturing and safety are coupled specifications: a tighter barrier improves immune exclusion while impairing oxygen delivery, and no single membrane optimises all simultaneously. Second, hypoimmune engineering now allows immune protection to be built into the cell product rather than supplied by the device. The question is therefore no longer whether encapsulation should continue, but which functions a biomaterial device performs better than the engineered cell—we argue containment, tissue integration and retrieval, not immunoisolation.

## 2 Why passive immunoisolation remains systemically constrained

The relevant benchmark is established islet transplantation under systemic immunosuppression, which can provide durable metabolic benefit and, in selected recipients, insulin independence (Shapiro et al., 2000; Hering et al., 2016; Lablanche et al., 2018; Marfil-Garza et al., 2022; Catarinella et al., 2025). Encapsulation must approach that efficacy while removing immunosuppression and ultimately overcoming donor scarcity. Graft survival, detectable C-peptide or transient reductions in exogenous insulin requirement are intermediate observations rather than therapeutic endpoints; human studies have repeatedly demonstrated viable, secreting grafts without a clinically sufficient metabolic effect (Tuch et al., 2009; Shapiro et al., 2021), indicating that biological activity should not be equated with therapeutic efficacy.

The difficulty is architectural. A membrane must exclude cellular and soluble immune effectors while admitting oxygen and nutrients and exporting insulin at physiologically relevant rates. Selectivity is multidimensional, but increasing barrier stringency generally raises device-level transport resistance and prevents direct vascular integration (Colton, 1995; Desai and Shea, 2017). The ViaCyte programmes provide an informative, although non-controlled, clinical contrast. The sealed VC-01/PEC-Encap device showed findings consistent with partial immune shielding, yet cell survival was inconsistent and was reported to be primarily limited by the foreign-body response to the device (Henry et al., 2018). Opening the architecture in VC-02/PEC-Direct restored vascular access but removed immunoprotection, requiring systemic immunosuppression; only 6 of 17 recipients became C-peptide positive and none achieved insulin independence (Ramzy et al., 2021; Shapiro et al., 2021). Clinical experience with these devices is summarised in Supplementary Table S1. The two configurations delimit the same constraint from opposite directions: the sealed membrane elicited a foreign-body response limiting oxygenation, whereas the open device restored vascular access but reinstated systemic immunosuppression. Both outcomes arise from the coupling between immune exclusion and mass transfer, and are therefore not resolved by substituting polymer chemistry.

Oxygenation is the least negotiable component of that constraint. β-cells are highly hypoxia-sensitive, and graft oxygen-consumption rate predicts functional performance (Dionne et al., 1993; Papas et al., 2007). Encapsulation replaces the intra-islet capillary network with passive diffusion. In avascular constructs, reaction-diffusion modelling and explant histology concur that beyond a modest depth oxygen tension falls below the β-cell threshold, producing a hypoxic core at the tissue densities a human dose requires (Buchwald, 2009; Suszynski et al., 2014; Iwata et al., 2018). The same transport limitation attenuates insulin egress, such that diffusion-only macrodevices may not reproduce the secretion kinetics required for physiological glucose control unless an additional driving force is introduced (Thomson et al., 2025). Each mitigation introduces a compensatory constraint. Active oxygenation can sustain human islets without immunosuppression but adds hardware and further failure modes (Ludwig et al., 2013; Carlsson et al., 2018; Krishnan et al., 2026); prevascularisation improves access to perfused tissue but recruits host vasculature to a membrane whose function requires excluding it (Papas et al., 2019). Each approach relaxes one requirement at the expense of another, characteristic of a coupled rather than independently optimisable specification. Scaling compounds these effects: therapeutic cell mass, surface-to-volume ratio, implant geometry, duration and the evolving foreign-body response jointly determine performance (Veiseh et al., 2015; Doloff et al., 2017; Bochenek et al., 2026). Human micro- and macroencapsulation studies, together with xenogeneic experience, have consequently established feasibility and persistence more consistently than durable metabolic efficacy (Soon-Shiong et al., 1994; Calafiore et al., 2006; Elliott et al., 2007; Basta et al., 2011; Jacobs-Tulleneers-Thevissen et al., 2013; Ludwig et al., 2017; Matsumoto et al., 2016).

Pluripotent stem-cell differentiation has largely removed cell supply as the conceptual bottleneck: β-like cells can be manufactured at scale and can restore glucose control in animals (Pagliuca et al., 2014; Rezania et al., 2014). Clinically, fully differentiated stem-cell-derived islets transplanted without a device but with immunosuppression have produced robust function, including insulin independence (Reichman et al., 2025). By contrast, durable efficacy from an immunosuppression-free encapsulated product remains unproven. Vertex discontinued VX-264 after its Day-90 efficacy endpoint was not met, a company-reported negative result that is consistent with, although does not mechanistically establish, limitations in engraftment and mass transport (Vertex Pharmaceuticals, 2025). Abundant cells therefore do not resolve device physics; they expose these constraints at therapeutic scale and add requirements for oncological containment and retrieval. Immunoisolation is therefore a means to a defined clinical outcome—transplantation without systemic immunosuppression—rather than a therapeutic objective in itself (Scharp and Marchetti, 2014; Desai and Shea, 2017), a distinction that acquires practical importance once that outcome becomes attainable by other means.

## 3 Reassigning immune protection to the cell product

Hypoimmune engineering changes the premise that immune protection must be supplied by the device. Disruption of B2M reduces HLA class I expression, disruption of CIITA limits inducible HLA class II expression, and complementary strategies such as CD47 overexpression or retention of non-polymorphic HLA molecules mitigate missing-self recognition (Deuse et al., 2019; Parent et al., 2021). Current implementations disrupt B2M, eliminating surface HLA class I and the CD8^+^ T-cell target, and CIITA, removing inducible HLA class II and the direct CD4^+^ T-cell target, while adding an inhibitory signal—commonly CD47 overexpression—to restrain the natural-killer-cell ‘missing-self’ response that HLA class I loss provokes (Deuse et al., 2019; Hu et al., 2024a). These interventions do not render a cell immunologically inert; they attenuate recognition at its source, relocating immune protection from a barrier around the cell to its genome. Immune-evasion strategies are compared in Supplementary Table S2. The argument does not depend on a single modification: selective deletion of polymorphic HLA with retention of non-polymorphic HLA-E, -F and -G implements the same principle (Parent et al., 2021). Residual recognition—innate and cytokine-mediated bystander injury, and indirect allorecognition of shed antigen by host antigen-presenting cells—remains MHC-independent and is addressed by neither (Bulek et al., 2012; Newby and Mathews, 2017). The cell products under evaluation span primary donor islets, stem-cell-derived islets and pancreatic endoderm (Pagliuca et al., 2014; Rezania et al., 2014; Ramzy et al., 2021; Reichman et al., 2025).

This strategy may address both alloimmunity and selected components of recurrent autoimmunity. Loss of HLA class I removes a central substrate for direct CD8-mediated β-cell recognition, whereas CIITA disruption constrains direct HLA class II-dependent recognition. Indirect antigen presentation by host antigen-presenting cells, innate sensing, NK-cell surveillance and cytokine-mediated injury remain possible (Bulek et al., 2012; Newby and Mathews, 2017). The translational evidence has advanced along a defined sequence: hypoimmune cells first evaded rejection in immunocompetent allogeneic hosts (Deuse et al., 2019), then persisted in immunocompetent macaques, and hypoimmune islets achieved insulin independence in an immunocompetent diabetic non-human primate without immunosuppression or detectable immune response (Hu et al., 2024a; Hu et al., 2024b). The most consequential observation followed in a first human recipient: gene-edited allogeneic islets implanted in forearm muscle, without any immunosuppressive drug, remained glucose responsive, with continued graft survival and immune evasion reported at 14 months and graft cells confirmed at the site by imaging (Carlsson et al., 2025; Carlsson et al., 2026).

This evidence remains limited in scope: a single participant, a single scientific lineage, and efficacy not yet established at therapeutic scale (Carlsson et al., 2025; Carlsson et al., 2026). Its qualitative implication is nonetheless robust: membrane-based immunoisolation is no longer the only demonstrated route to graft survival without systemic immunosuppression.

The implication is functional rather than semantic. Immunoisolation is valuable because it seeks freedom from immunosuppression; it is not itself the therapeutic endpoint. If cell engineering can deliver that outcome without the transport penalty and foreign-body interface a closed barrier imposes, passive immunoisolation may no longer be the preferred primary design objective—it would, for its founding purpose, be competing against an approach avoiding several of its principal device-level costs. This is presented as a direction supported by current evidence rather than an established conclusion, contingent on independent replication across cell products and recipients, durability, control of residual immune-escape routes, and long-term safety. Where editing is unavailable—xenogeneic or non-editable products—membrane immunoisolation retains a clear rationale.

## 4 A new biomaterials brief: containment, integration and retrieval

The novelty is not a new material but a new specification: the device’s hardest historical constraint—molecular selectivity sufficient to exclude antibodies and complement—becomes negotiable once the cell carries its own immune protection, leaving a different and more tractable problem, containment. Once immune protection is assigned substantially to the cell product, the device-specific rationale becomes physical safety: an immune-evasive graft may be less readily eliminated if it acquires an undesirable phenotype, and pluripotent-derived products retain residual risks of proliferative or off-target cells. A favourable anatomical site improves surveillance and surgical access, as illustrated by the forearm-muscle implantation used in the first human hypoimmune-islet study (Carlsson et al., 2025), but does not ensure that the graft remains spatially bounded. Anatomical accessibility does not ensure recoverability: an unconfined proliferative graft may disperse along tissue planes, and cells integrated diffusely into skeletal muscle cannot be excised as a discrete unit. A containment platform provides what site selection cannot—restricted cell migration and a defined explant procedure in place of open-ended excision—with the objective of demonstrably complete graft recovery, a requirement heightened for immune-evasive cells that are by design less susceptible to host-mediated elimination.

This brief replaces a high-selectivity immunoisolating architecture with a low-transport-resistance, defect-controlled and cell-retaining one (Figure 1). The relevant material classes differ accordingly: immunoisolation has relied on crosslinked, chemically modified alginates and selectively permeable membranes (Calafiore et al., 2006; Vegas et al., 2016a), whereas containment admits nanofibrous and macroporous architectures in which retention derives from fibre and pore geometry rather than molecular sieving (Wang et al., 2021). The distinction is not reducible to nominal pore size. Immunoisolation must manage cells, immunoglobulins, complement and soluble inflammatory mediators while maintaining metabolic exchange; containment must prevent migration of whole graft cells while minimising resistance to oxygen, glucose, insulin and other relevant solutes. A nanofibrous device in which fibre dimensions below approximately 500 nm prevented cell penetration while preserving mass transfer illustrates a containment-relevant membrane principle (Wang et al., 2021).

**FIGURE 1:**
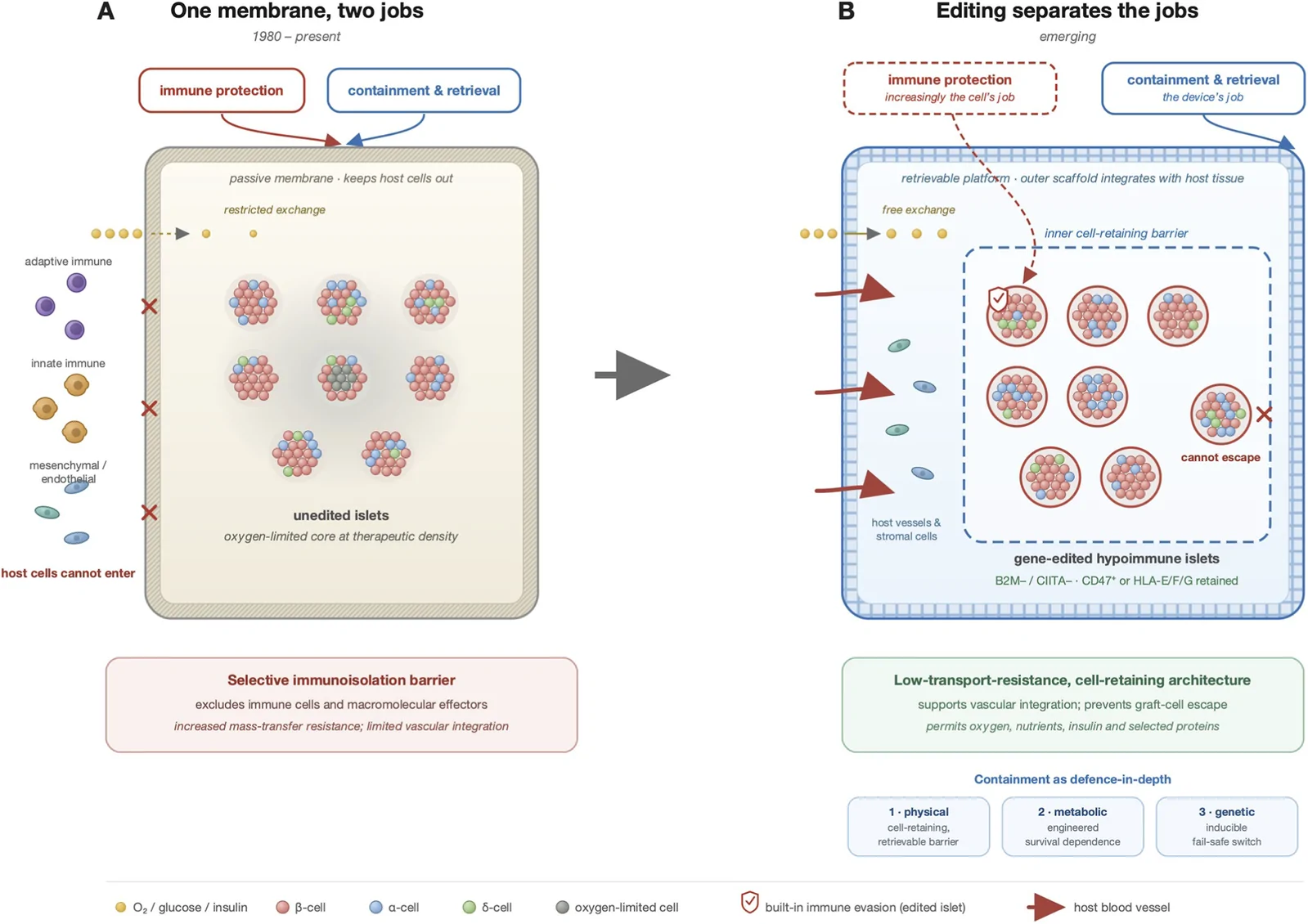
Disaggregating the capsule's two jobs, and the membrane regime that follows. **(A)** 1980–present: a single passive membrane is asked to perform two functions at once—immune protection (exclude lymphocytes, immunoglobulin, complement and cytokines) and containment/retrieval confine the graft, allow removal, and prevent escape)-while. graft, allow removal, prevent escape) — while also transporting oxygen and nutrients to unedited cells; at therapeutic cell density the resulting diffusion limitation produces an oxygen-limited islet core (grey cells). Islets are drawn as multicellular aggregates of β-, α- and δ-cells. A barrier built to be immunoisolating raises total mass-transfer resistance and precludes direct vascular integration, so immune protection and physiological transport stay coupled at the device level and the design is over-constrained; host vascularisation is largely excluded because vessels would also carry immune access. **(B)** Emerging: gene-edited hypoimmune cells (B2M and CIITA disruption with CD47 overexpression or retention of non-polymorphic HLA-E, -F and -G; shield glyph) attenuate immune recognition at the level of the cell—shown in immunocompetent primates and, so far, one human recipient (Carlsson et al., 2025; Carlsson et al., 2026; Deuse et al., 2019; Hu et al., 2024a; Hu et al., 2024b). This can relieve the device of its immunological role, leaving containment, retrievability and safety. The requirement inverts: rather than excluding molecules, the device must retain whole cells (islets ∼100–200 µm) while oxygen, glucose, insulin and relevant proteins move with comparatively low resistance—a high-selectivity immunoisolating architecture giving way to a low-transport-resistance, defect-controlled, cell-retaining one. Directionality (host cells in, graft cells confined) does not follow from porosity alone, since host and graft cells are of comparable size; a plausible construction couples an external vascularising scaffold, into which host vessels and stroma integrate, with an internal cell-retaining barrier that confines the graft (dashed boundary). Containment could be reinforced as defence-in-depth (lower right): a physical, cell-retaining, retrievable barrier (Wang et al., 2021), engineered metabolic survival-dependence (Rovner et al., 2015), and a genetic fail-safe (Izadifar et al., 2024). The figure’s message: editing does not make the capsule obsolete—it clarifies which of its two jobs is actually its own. This is a conceptual schematic; the containment device is a target product profile, not a clinically validated object, and the two-compartment design is illustrative rather than an existing device.

At the transport level, “permeability” must be used precisely. A material’s intrinsic transport property—how readily a solute dissolves into and diffuses through it—is distinct from the transport delivered by a finished device, which also depends on membrane thickness and, in porous constructs, on the open fraction, interconnectivity and tortuosity of the wall. Specifying the material alone therefore says little about device performance. Oxygen is the binding constraint, and whether a design will be oxygen-limited can be predicted before fabrication by weighing cellular oxygen consumption against resupply across the distance to the nearest perfused vessel—beyond a threshold cell density or diffusion distance, a hypoxic core becomes unavoidable (Buchwald, 2009; Papas et al., 2019; Orimi et al., 2025). Qualification thresholds for transport, containment, mechanics, interface and manufacturing are set out in Table 1.

**TABLE 1:** A translational stress test—falsifiable go/no-go criteria before clinical entry.

Domain	Critical question (falsifiable)	Required evidence	Reason to stop development
Dose, geometry and oxygenation	Can the device hold the full clinical dose and oxygenate it without a hypoxic core?	Reaction–diffusion model integrating cell-specific O_2_ consumption, effective diffusivity, barrier and fibrotic resistance, loading density and distance from perfused microvasculature; validated by spatial O_2_ and viability mapping	The therapeutic dose is not implantable, or modelling and histology predict an unavoidable hypoxic core
Large-animal validation	Does it work in an immunocompetent large animal at translatable dose?	Sustained function at clinically relevant scale and duration in an immunocompetent large animal	Function only in rodents or immunodeficient hosts
Durability and foreign-body response	Does function persist for years despite the foreign-body response?	Longitudinal fibrosis, perfusion and graft-function assessment over the intended implant duration	Function is short-lived or progressively extinguished by the mature foreign-body response
Barrier and mechanical integrity	Does the barrier stay intact under load over the implant lifetime?	Defect mapping plus geometry-appropriate creep, fatigue, puncture, delamination, seam-integrity and leak testing before and after sterilisation, ageing and chronic implantation	Defect propagation, leakage or mechanical failure within the intended lifetime
Containment and complete retrieval	Can an immune-evasive, proliferative graft be fully bounded and retrieved after chronic implantation?	Cell-escape testing under pressure and cyclic deformation; seam and port integrity; complete explantation verified by imaging, histology and product-specific molecular biodistribution	Cells escape, residual graft is detected, or the device cannot be removed intact
Manufacturing and potency	Does performance survive component sterilisation, aseptic fill-and-seal, storage and transport?	Critical material attributes linked to process parameters; sterility assurance; residuals, extractables/leachables and endotoxin; lot-to-lot permeance, mechanics, stability and post-loading potency	Processing changes performance or no release assay predicts in-vivo function
Host–material interface	Does tissue integration preserve vascular proximity without compromising retrieval or transport?	Protein adsorption and complement; macrophage/giant-cell response; perfusion, vessel-to-graft distance, local oxygen transfer, fibrotic thickness and chronic permeance	Fibrotic separation, loss of perfusion, altered permeance or tissue incorporation that precludes complete retrieval
Oncologic safety	Is a proliferative cell source safe and containable?	Duration-matched tumorigenicity, off-target growth and containment studies	Detectable proliferative or off-target risk
Efficacy and allocation of immune protection	Is the metabolic effect clinically meaningful, and is immune protection assigned to the cell (editing) or the device (membrane)?	Prespecified clinical endpoint versus comparator, plus immune-evasion evidence or a product-specific rationale for membrane immunoisolation	The effect is clinically insufficient, or the device is assigned immune protection already delivered by the cell product

Containment is a distributional and mechanical specification rather than a mean-pore-size threshold: it depends on the upper tail of the pore-size distribution, manufacturing defects, and the integrity of seams, welds and filling ports (Wang et al., 2021), and must be demonstrated by escape testing under physiological load rather than assumed from nominal porosity. Retrieval constitutes a material-performance endpoint: retrievability is determined by material and structural properties as well as surgical approach, and should be verified by explantation without fragmentation together with product-specific molecular biodistribution assays rather than device inspection alone.

The host-material interface remains a primary determinant of long-term function: protein adsorption, complement activation, macrophage and foreign-body giant-cell responses and collagen deposition progressively alter vascular proximity, transport resistance and explant mechanics (Veiseh et al., 2015; Doloff et al., 2017). The relevant endpoint is not absence of fibrosis but preservation of perfusion, permeance and retrievability after a mature host response. A plausible architecture separates these requirements spatially: an external scaffold with interconnected macroporosity supports perfused vascular and stromal integration, while an internal defect-controlled barrier confines the graft. The interface between layers becomes a critical failure mode, requiring assessment for delamination, fluid accumulation, fibrotic separation and loss of vascular proximity. The Cell Pouch provides a clinical example of a retrievable, prevascularised tissue chamber, although its current donor-islet programme still uses systemic immunosuppression (Pepper et al., 2015; Sernova Biotherapeutics, 2019). Physical containment may also be reinforced by engineered metabolic dependence and inducible genetic fail-safes, creating a defence-in-depth strategy rather than reliance on a single barrier (Rovner et al., 2015; Izadifar et al., 2024).

These attributes must be maintained through component sterilisation, aseptic loading, sealing, storage and transport (Table 1), with potency defined rather than inferred (Pipeleers et al., 2016; Robert et al., 2019): a device meeting specification before filling but not after has not met its target product profile.

Two further directions merit consideration as working hypotheses, both reframing containment as co-engineered between material and cell rather than enforced by a barrier alone. The first is biological anchoring: designing the internal surface and graft so that cells adhere and home to the device—via cell-adhesion ligands or extracellular-matrix-mimetic coatings—such that retention derives from adhesive preference as well as pore geometry. The second converts the fail-safe from a lethal to a spatial mechanism: rather than a globally inducible suicide switch, a graft could be made dependent for survival on a factor supplied only within the compartment, extending engineered metabolic dependence (Rovner et al., 2015), so that escaping cells lose viability without further intervention. Both remain speculative and carry identifiable risks, including adhesion sufficient to impede retrieval and loss of spatial specificity where the required factor diffuses beyond the compartment. They nonetheless illustrate a general point: once relieved of the immunoisolation requirement, containment becomes a multi-parameter design problem rather than a single barrier specification.

## 5 Discussion

The translational consequence reverses the conventional development sequence (Colton, 1995; Papas et al., 2007). Rather than selecting a material, demonstrating short-term rodent compatibility and deferring scale, oxygenation and retrieval to later stages, development should begin with the indication and target product profile. Therapeutic dose, durability, metabolic endpoint, oxygen model, host-interface requirements, containment and explant strategy should be fixed before geometry and material selection. Elements of this discipline exist in isolation—physics-based oxygen modelling constraining dose and geometry, potency defined as functional β-cell mass, oxygen-supported devices in clinical testing—but are rarely imposed together before a material is selected. The proposed stress test (Table 1) operationalises this approach: each domain is phrased as a question that can be failed, with the evidence required to satisfy it and the result that halts development specified in advance, so that termination becomes a pre-agreed outcome rather than a late and costly one.

The proposition is falsifiable: it would be substantially weakened if hypoimmune engineering failed to provide durable protection (Deuse et al., 2019; Hu et al., 2024a) across independent products and recipients; if passive membrane immunoisolation achieved durable insulin independence without immunosuppression; or if a therapeutic dose of edited cells could not be contained and completely retrieved. Until those data emerge, the evidence supports progressively reallocating immune protection to the cell product while assigning the device to containment, integration and safety.

This does not invalidate the broader encapsulation field. Membrane immunoisolation remains relevant for xenogeneic or non-editable products, and local immunomodulation, fibrosis-resistant chemistries, controlled oxygenation, prospective transport modelling and potency defined as functional β-cell mass rather than inferred all remain valuable across device classes (Vegas et al., 2016a; Vegas et al., 2016b; Pipeleers et al., 2016; Robert et al., 2019; Grimus et al., 2024; Bochenek et al., 2026; Krishnan et al., 2026). What should be retired is the assumption that viability, detectable secretion or rodent efficacy are adequate surrogates for a clinically meaningful, scalable and retrievable graft.

The persistent difficulty of islet encapsulation has not been the absence of an optimal polymer, but the concentration of incompatible functions within a single passive boundary (Colton, 1995; Desai and Shea, 2017; Grimus et al., 2024). Hypoimmune engineering begins to separate those functions. Its emergence does not remove the need for biomaterials; it makes their purpose more precise. The next generation of devices should be judged not by how completely they reproduce the immunological ambition of 1980, but by whether they can support a therapeutic cell mass, integrate with perfused tissue, prevent escape and permit complete retrieval over the intended lifetime. On this reading the device is not obsolete but redefined: an immune-evasive, proliferative cell product is precisely the graft for which a retrievable containment layer is most necessary, and designing that layer is a more demanding biomaterials problem than the one originally assigned to the capsule. The capsule was never a single-function object; it addressed two design problems that had not been distinguished, and the migration of immune protection into the cell product now permits the second to be specified in its own terms.

The framework is a proposal (§5); individual domains draw on the evidence cited for oxygen and transport (Buchwald, 2009; Papas et al., 2007; Iwata et al., 2018; Papas et al., 2019; Orimi et al., 2025), foreign-body response (Veiseh et al., 2015; Doloff et al., 2017), potency (Pipeleers et al., 2016; Robert et al., 2019), clinical benchmarks (Hering et al., 2016; Lablanche et al., 2018; Marfil-Garza et al., 2022; Catarinella et al., 2025) and hypoimmune engineering (Deuse et al., 2019; Hu et al., 2024a; Hu et al., 2024b; Carlsson et al., 2025). It is intended as a stage-proportionate set of preclinical-entry questions rather than an absolute gate. The containment, host-interface and retrieval domains apply specifically to the target product profile proposed in §4.

## Data Availability

The original contributions presented in the study are included in the article/Supplementary Material, further inquiries can be directed to the corresponding author.
