# Supplementary material for: The capsule was never just a capsule: what islet encapsulation is for, now that immunoisolation is no longer its job
Source: Front Bioeng Biotechnol. 2026 Oct 8;14:1943061. doi: 10.3389/fbioe.2026.1943061 (PMC13687413; doi:10.3389/fbioe.2026.1943061)
Supplement: Supplementary file 1 [file Table1.docx]

# Supplementary Table S1 Clinical experience with immunoisolation and containment devices in β-cell replacement

Studies are ordered by device class. "Immunosuppression" indicates whether systemic immunosuppression was administered as part of the protocol. Outcomes are reported as published or as disclosed by the sponsor; where a programme was discontinued, wording follows the sponsor's public statement.

| **Device / programme** | **Class** | **Cell source** | **Registration** | **n** | **Immunosuppression** | **Principal reported outcome** | **Source type** |
| --- | --- | --- | --- | --- | --- | --- | --- |
| **Alginate microcapsules (Soon-Shiong)** | Micro- encapsulation | Human donor islets | Not registered | 1 | Yes (pre-existing) | Insulin independence reported in a single recipient with a functioning renal allograft | Peer-reviewed (Soon-Shiong et al., 1994) |
| **Alginate microcapsules (Calafiore/Basta)** | Micro- encapsulation | Human donor islets | Not registered | 4 | No | Graft survival with C-peptide, without insulin independence | Peer-reviewed (Basta et al., 2011) |
| **Alginate microcapsules (Tuch)** | Micro- encapsulation | Human donor islets | Not registered | 4 | No | Viable, secreting grafts without clinically sufficient metabolic effect | Peer-reviewed (Tuch et al., 2009) |
| **Alginate macrobeads (Jacobs-Tulleneers-Thevissen)** | Micro- encapsulation | Human donor islet cells | Not registered | 1 | No | Sustained graft function without insulin independence | Peer-reviewed (Jacobs-Tulleneers-Thevissen et al., 2013) |
| **Encapsulated porcine islets (Elliott)** | Micro- encapsulation, xenogeneic | Neonatal porcine islets | Not registered | 1 | No | Encapsulated cells recovered viable at 9.5 years | Peer-reviewed (Elliott et al., 2007) |
| **βAir (Beta-O₂)** | Macro- encapsulation, oxygen-supplied | Human donor islets | - | 4 | No | Graft survival with preserved C-peptide; no insulin independence | Peer-reviewed (Ludwig et al., 2013; Carlsson et al., 2018) |
| **VC-01 / PEC-Encap (sealed)** | Macro- encapsulation, immunoisolating | hESC-derived pancreatic endoderm | NCT02239354 | 19 | No | Cell survival to 24 months; efficacy limited by a foreign-body response to the device; sub-therapeutic dose cohort | Conference abstract (Henry et al., 2018) |
| **VC-02 / PEC-Direct (open, non-immunoisolating)** | Macro- encapsulation, vascularising | hESC-derived pancreatic endoderm | NCT03163511 | 17 | Yes | Meal-regulated C-peptide in a minority of recipients (6/17); no insulin independence | Peer-reviewed (Ramzy et al., 2021; Shapiro et al., 2021) |
| **VX-264** | Macro- encapsulation, immunoisolating | Stem-cell-derived islets | NCT05791201 | 7 | No | Discontinued; per the sponsor, did not achieve clinically meaningful insulin production | Company communication (Vertex Pharmaceuticals, 2025) |
| **Cell Pouch (Sernova)** | Prevascularised containment chamber | Human donor islets | NCT03513939 | 17* | Yes | Retrievable prevascularised chamber; programme ongoing with systemic immunosuppression | Registry (Sernova Biotherapeutics, 2019); peer-reviewed preclinical (Pepper et al., 2015) |
| **UP421 (hypoimmune islets; no device)** | Cell-intrinsic protection | Gene-edited allogeneic donor islets | NCT06239636 | 1 | No | Graft survival and immune evasion at 12 weeks, extended to 14 months; glucose-responsive C-peptide | Peer-reviewed (Carlsson et al., 2025; Carlsson et al., 2026) |
| **Zimislecel (formerly VX-880; no immunoisolating device)** | Free islet infusion | Stem-cell-derived islets | NCT04786262 | 12 | Yes | Insulin independence in the majority of recipients at one year | Peer-reviewed (Reichman et al., 2025) |

*Note. *Planned enrolment for the Cell Pouch study. The final two rows are included as comparators: they are not encapsulation studies, but establish (i) that freedom from immunosuppression has been achieved without a membrane, and (ii) that clinically meaningful efficacy has been achieved with immunosuppression and no device. Together they define the two reference points against which any future containment device must be justified.*

*References cited in this table appear in full in the main reference list.*
