# Supplementary material for: The capsule was never just a capsule: what islet encapsulation is for, now that immunoisolation is no longer its job
Source: Front Bioeng Biotechnol. 2026 Oct 8;14:1943061. doi: 10.3389/fbioe.2026.1943061 (PMC13687413; doi:10.3389/fbioe.2026.1943061)
Supplement: Supplementary file 2 [file Table2.docx]

# Supplementary Table S2 Immune-evasion strategies referenced in the main text

| **Modification** | **Immunological target removed or engaged** | **Residual exposure** | **Reference** |
| --- | --- | --- | --- |
| **B2M disruption** | Surface HLA class I; direct CD8⁺ T-cell recognition | Natural-killer-cell 'missing-self' activation | Deuse et al., 2019 |
| **CIITA disruption** | Inducible HLA class II; direct CD4⁺ T-cell recognition | Indirect allorecognition via host antigen-presenting cells | Deuse et al., 2019; Hu et al., 2024a |
| **CD47 overexpression** | Engages SIRPα; inhibits NK and macrophage killing | Does not address cytokine-mediated bystander injury | Deuse et al., 2019; Hu et al., 2024a |
| **Selective HLA deletion with HLA-E/-F/-G retention** | Polymorphic HLA removed; non-polymorphic ligands retained for NK inhibition | MHC-independent innate injury | Parent et al., 2021 |
| **Engineered metabolic dependence** | Not immunological; restricts survival to a supplied factor | Loss of specificity if the factor diffuses | Rovner et al., 2015 |
| **Inducible genetic fail-safe** | Not immunological; permits ablation on demand | Requires a functional switch in all cells | Izadifar et al., 2024 |

*References cited in this table appear in full in the main reference list.*
